# Sample Injection for Real-Time Analysis (SIRTA) Using GC-MS with Cold EI

**DOI:** 10.1021/jasms.3c00412

**Published:** 2024-01-18

**Authors:** Oneg Elkabets, Benny Neumark, Aviv Amirav

**Affiliations:** † School of Chemistry, Tel Aviv University, Tel Aviv 6997801, Israel; ‡ Aviv Analytical Ltd, 24 Hanagar Street, Hod Hasharon 4527713, Israel

**Keywords:** SIRTA, real-time analysis, GC-MS with Cold EI, flow injection

## Abstract

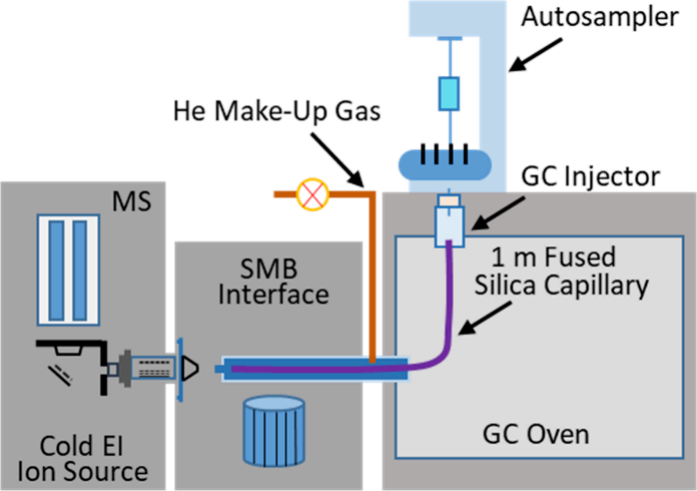

There is a continual demand for advanced methods and instruments for real-time analysis (RTA). Most of the current RTA techniques based on MS involve ambient desorption ionization technology. However, flow injection of liquid extracted samples is another option without added modifications or cost to existing LC-MS instruments. In this work, we introduce a new RTA approach named sample injection for real-time analysis (SIRTA) using GC-MS with Cold EI. In SIRTA, the standard GC column is replaced with a 1 m long 0.1 mm I.D. fused silica capillary that connects the GC injector to the MS transfer-line of Cold EI. Thus, SIRTA with Cold EI imposes no need for any additional instrumentation; hence, it is characterized by zero added cost. Like in flow injection in MS of LC-MS, the sample is dissolved in ∼1 mL methanol or another solvent. Subsequently, the vial is placed in the GC-MS autosampler while using a standard syringe for injection without any GC separation. The analysis takes merely 0.2–0.7 min, ensuring rapid and consecutive analyses. Unlike standard EI, Cold EI enables SIRTA by taking advantage of its fly through open ion source to avoid overwhelming the ion source during the elution of solvents while still providing enhanced molecular ions for nearly all analytes. In this study, we demonstrated SIRTA Cold EI analysis of 12 compounds and 7 mixtures, including various prescription and illicit drugs, cannabis and petroleum samples, and other synthetic organic compounds including those with molecular weight up to 800 g/mol.

## 1 Introduction

Real-time analysis (RTA) is a widely explored topic in the mass spectrometry (MS) field. The goal of RTA is to shorten the full analysis time to <1 min including sample handling and preparation. Among the various RTA methods, ambient desorption ionization (ADI) is a popular and widely explored research topic. There are 85 different ADI methods and instrumentations as described in the literature.^1^ Among the various ADI methods, the most common are direct analysis in real-time (DART),^2^ desorption electrospray ionization (DESI),^3^ atmospheric solids analysis probe (ASAP),^4^ and open port sample introduction^5−7^ as recently reviewed.^8^ The various ADI methods and instruments allow fast analysis of powders and organic surfaces without sample preparation through ambient (atmospheric) pressure desorption and ionization followed by ion transfer into the mass spectrometer through an ion funnel as used in LC-MS systems. Whereas ADI provides one form of RTA, probably the first report of RTA using MS was in 1979 by Lovett et al. involving APCI-MS for breath analysis.^9^

However, a traditional, simple, and well-established flow injection analysis is available in every LC-MS system simply by removing the LC column and replacing it with a low dead volume LC union. Flow injection provides an accessible alternative to other RTA methods for sample analytes that are dissolved in liquids or via simple sample solvation. Solid samples such as powders can be placed by touching their powder on a thin glass rod or a melting point vial as in ASAP and then dipping it in a 1 mL vial with 0.5–1 mL of methanol for syringe-based flow-injection analysis. Unlike ADI methods, automated flow-injection analysis does not require modifications or additional costs using existing LC-MS instrumentation. In fact, a syringe pump could replace the LC to lower the cost for RTA by flow-injection analysis.

While flow injection is an option using LC-MS systems, it is not possible in traditional GC-MS using standard electron ionization (EI) ion sources. The solvent elution leads to overwhelming intraion-source space charge effects and could likely damage the filament. Thus, GC-MS with standard EI systems requires a “solvent delay” during solvent elution times with the filament turned off.

However, alternate forms of ionization in MS of GC-MS can avoid the need for the solvent delay. For example, an early report of RTA was published in 1998 using laser desorption ultrafast GC-MS with supersonic molecular beams.^10^ The laser desorbed organic compounds from a variety of surfaces at ambient pressure without sample preparation followed by helium sweeping and ultrafast GC separation in a matter of seconds with MS analysis by in-vacuum EI or hyperthermal surface ionization. Direct sample introduction (DSI) is another approach to RTA.^11^ It was commercialized by Varian and later by Bruker by the name ChromatoProbe and by FLIR, and Agilent sells it today by the commercial name of Thermal Separation Probe. Unlike the standard MS probe analysis that typically takes >5 min, the ChromatoProbe enables near RTA in 1–2 min without sample preparation.^11^ In 2010, the Open Probe method and device were described.^12^ Open Probe enables RTA in a matter of few seconds without sample preparation. Subsequently, Open Probe Fast GC-MS was developed^13,14^ (with standard EI or Cold EI), which provides RTA with ultrafast GC separation and library identification including at the isomer level (available by Agilent under the name QuickProbe^15^).

While these methods and instruments are very effective in RTA analysis with MS using vacuum ion sources, they still require additional hardware and cost. In this work, we avoid these needs by introducing sample injection for real-time analysis (SIRTA) that does not require added hardware and cost while being used in GC-MS with Cold EI.

GC-MS with Cold EI is based on coupling of the GC and MS with a supersonic molecular beam (SMB) interface and on electron ionization of vibrationally cold sample molecules during their flight through a contact-free ion source (thereby named Cold EI). Cold EI was developed in 1990 by Amirav and his group^16,17^ and has been reviewed.^18,19^ Also, a book was recently published on GC-MS with Cold EI.^20^ Cold EI improves all the major GC-MS performance aspects including the following: (a) enhanced molecular ions while retaining NIST library identification; (b) significant extension of the range of compounds that are amenable for GC-MS analysis;^21^ (c) uniform response to all analytes for improved internal quantification; (d) faster analysis; (e) greater selectivity; (f) lower limits of detection.^22^ The aim of this paper is to describe the application of SIRTA with Cold EI after minimal or no sample preparation.

## 2 Experimental Section

The GC-MS with Cold EI system that was used is based on the combination of an Agilent 7890A GC + 5977 MSD (Agilent Technologies, Santa Clara, CA) with the Aviv Analytical Cold EI (Aviv Analytical LTD, Hod Hasharon, Israel). Cold EI has been reviewed^18−21^ and further described.^21,22^ In Cold EI, the GC column flow output is mixed with helium makeup gas (∼50–60 mL/min total column and makeup flow rate at stabilized nozzle backing pressure), in front of a supersonic nozzle at the end of a temperature-controlled transfer line. The helium makeup gas can be mixed with perfluorotributylamine (PFTBA) for system tuning and mass calibration. Sample compounds in the helium gas expand from a 100 μm supersonic nozzle into a vacuum chamber that is pumped by a Varian Navigator 301 turbo molecular pump (Varian Inc., Torino Italy). The supersonic expansion vibrationally cools the sample compounds, and the supersonic free jet is skimmed and collimated by a 0.8 mm skimmer in a second vacuum chamber (pumped by the Agilent 5977 system “Performance” turbo molecular pump), where an SMB is formed. The vibrationally cold sample compounds in the SMB fly through a dual-cage EI ion source^23^ where they are ionized by 70 eV electrons with 1 mA emission current in the SIRTA experiments. The Cold EI ion source was used for 9 years without any service, as it is highly rugged. The ions that are formed are focused by an ion lens system, deflected 90° by an ion mirror, and enter the Agilent 5977 MS for their mass analysis and detection by the Agilent triple-axis ion detector while the data are processed by the Agilent ChemStation software.

As briefly shown in the graphical abstract, the GC (Agilent 7890A) was equipped with an autosampler that typically injected 1 μL sample solution in splitless mode from a standard 1 mL vial into a split/splitless injector. A 1 m fused silica capillary with 0.1 mm I.D. (Agilent Technologies part number 160-2635-1 and price of $30) connected the standard split/splitless GC injector to the Cold EI MS transfer line that ended in the SMB interface vacuum chamber, and it was operated with 2.2 mL/min typical capillary flow rate (in the range of 1–4 mL/min). The GC oven and transfer line were maintained at a 250–320 °C temperature range depending on the application. The Cold EI ion source was operated with a SIRTA tune method that was very similar to typical Cold EI operation tune but with a lower emission current of 1 mA instead of the typical 6 mA to better preserve filament operation during solvent elution. Tune was performed with PFTBA and 1 μL splitless methanol injection during its about 1 min elution at 2.2 mL/min capillary flow rate. We used methanol, hexane, and CDCl_3_ as solvents but can likely use any other solvent except acetonitrile that can damage the tungsten filament. The helium makeup gas flow rate was about 50 mL/min, and the SIRTA with Cold EI sample compound cooling was a little less than without solvent elution due to the solvent minor effect on the SMB cooling.

The Cold EI fly through ion source has a long (30 mm) filament and large ionization volume to reduce intraion-source space charge. In addition, during solvent elution time, the jet separation efficiency is automatically reduced and thus less solvent passes the skimmer and enters the ion source. Furthermore, we reduced the filament emission current from typically 6 mA to 1 mA. Thus, the combination of high ionization volume, a small portion of the solvent in the SMB, and low emission current fully eliminated the need of solvent delay as employed in standard EI.

## 3 Results and Discussion

**Figure 1:**
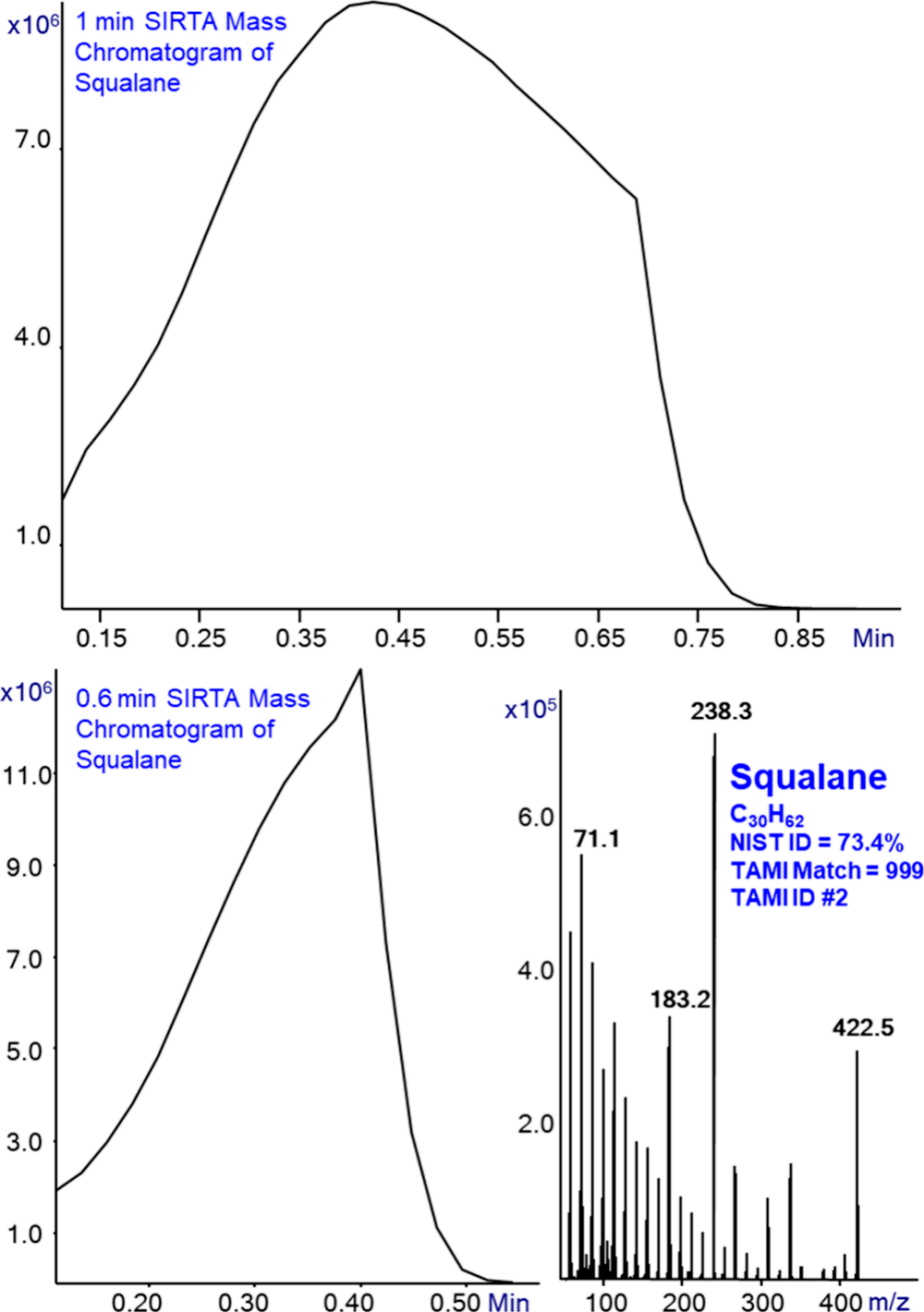
SIRTA Cold EI mass chromatogram of 1 μL of squalane in 1 min (upper) and in 0.6 min (bottom left) total analysis time and the squalane mass spectrum with its NIST and TAMI identification factors. The analysis was performed with 300 °C inlet, GC oven, and Cold EI transfer line temperatures.

Figure 1 shows the data obtained from a SIRTA of squalane (branched C_30_H_62_ hydrocarbon) with Cold EI. Squalane thick liquid was touched by a thin glass rod that was later dipped in a 1 mL vial with about 0.7 mL of methanol to provide about a few hundred ppm up to 0.1% squalane in methanol. This vial was placed in the GC autosampler and a 1 μL sample was injected splitless to obtain the mass chromatogram shown in the upper trace of Figure 1. The flow rate was 2.2 mL/min, and after 0.7 min the split was turned on with a split ratio of 10 (20 mL/min). As shown, the signal zeroed at about 0.9 min and the analysis took less than 1 min. As further shown in Figure 1, the mass chromatogram started at ∼0.1 min, as this is the time that it takes the Agilent ion detector voltage to turn on. The sample signal initially rises, as it takes a few seconds for the vaporized sample and solvent plug to reach the bottom of the injector liner, and it apexed at about 0.4 min and started to decline due to the gradual removal of the sample vapor from the GC injector liner. Obviously, this rate can be controlled by the capillary flow rate, and after exploring the range of 1 to 4 mL/min, we decided to use 2.2 mL/min as the optimal flow rate.

Figure 1 bottom left mass chromatogram shows the same squalane SIRTA analysis with the change that the split valve was opened at 0.4 min and thus the SIRTA analysis time was reduced to 0.6 min (36 s). While the shorter 0.6 min SIRTA analysis is better for sample identification the longer 1 min SIRTA analysis is preferable when SIRTA is used for mass spectral studies such as (a) the effect of electron energy in obtaining low eV soft Cold EI, (b) Cold EI tune at high masses beyond *m*/*z* = 503 of PFTBA such as with n-C_20_F_42_, and (c) MS/MS collision-induced dissociation (CID) energy study and optimization in Cold EI systems with MS/MS capability. Figure 1 bottom right trace shows the obtained Cold EI mass spectrum of squalane. Unlike in standard EI, we observe an abundant molecular ion and several isomer structurally informative fragment ions in Cold EI. We note that in standard EI the molecular ion abundance is <0.01%^24^ and the structurally informative ions are much weaker. However, despite the large enhancement of the molecular ion, the NIST identification probability was high at 73.4% (versus 37.4% in standard EI^24^) since the enhanced molecular ion usually improves the NIST identification probabilities.^24,25^ The NIST identification was further confirmed with our Tal-Aviv Molecule Identifier (TAMI) software^26,27^ that converts the experimental isotope abundances into elemental formulas with the limited mass accuracy of quadrupole mass analyzers. The TAMI software confirmed the NIST identification of C_30_H_62_ with a very high match factor of 999, and the elemental formula C_30_H_62_ was rated as its #2 in its generated table of elemental formulas while the mass spectrum clearly indicated squalane among the few initial options. We mention here the use of TAMI to show that SIRTA with Cold EI can provide elemental formulas like for the various ADI methods with high-resolution MS of LC-MS and yet its unique NIST library search and identification capability provides identification at the isomer level.

**Figure 2:**
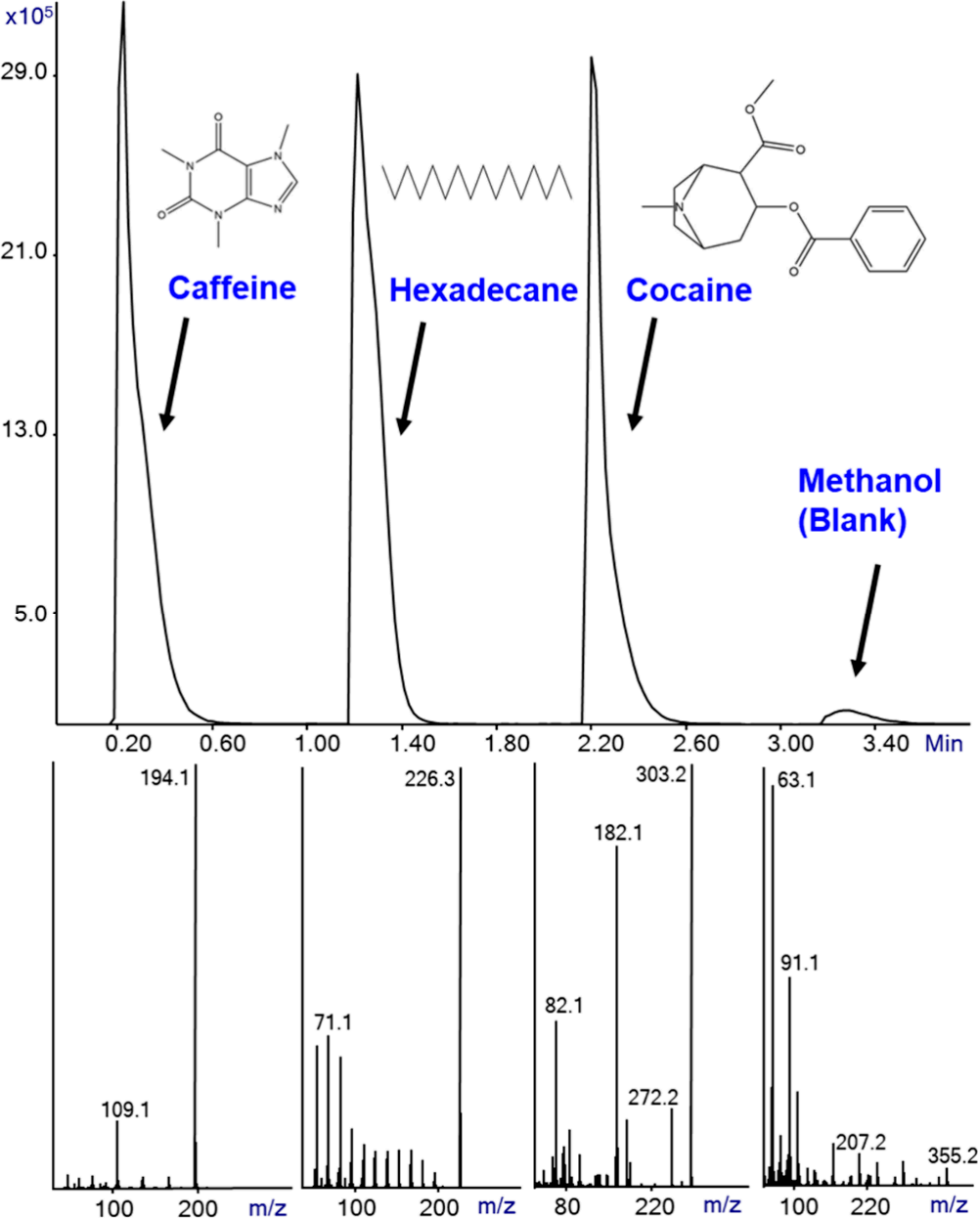
SIRTA Cold EI mass chromatogram (upper) of about 400 μg/mL each of caffeine, hexadecane, cocaine, and methanol impurities in one file using four manual injections, where each analysis time was 1 min. The bottom trace shows the generated caffeine, hexadecane, cocaine, and methanol Cold EI mass spectra. The analysis was performed with 300 °C inlet, GC oven, and transfer line temperatures.

The SIRTA Cold EI analysis of squalane as shown in Figure 1 was obtained with the standard Agilent autosampler injection that generates a separate file for its MassHunter or ChemStation based data analysis; autosampler preparations for the next analysis can take 30–40 s, and thus the full analysis cycle time is 1.5 min. In order to reduce the analysis time and enable several sample analyses in one “running” file we have to adopt manual injection as demonstrated in Figure 2.

Figure 2 shows the SIRTA mass chromatogram (upper) of about 400 μg/mL each caffeine, hexadecane (n-C_16_H_34_), cocaine, and methanol for its impurities content in one file using manual injections, where each sample analysis time was 1 min. The bottom trace shows the generated Cold EI mass spectra of the above-mentioned compounds. The analysis was performed with a 300 °C inlet, GC oven, and transfer line temperature, and the capillary flow rate was 2.2 mL/min combined with split ratio of 4 in the injections to reduce the analyses times. As shown, the Cold EI mass spectra exhibit largely enhanced molecular ion for hexadecane, some enhancement for cocaine, and minor enhancement for caffeine, yet all of them were easily identified by their NIST library searches with high identification probabilities. Methanol shows impurities at the few μg/mL levels plus some PDMS-related ions (*m*/*z* = 207, 281, and 355) probably due to septa pieces in the injector liner. Note that SIRTA merely took 1 min full analysis cycle time.

**Figure 3:**
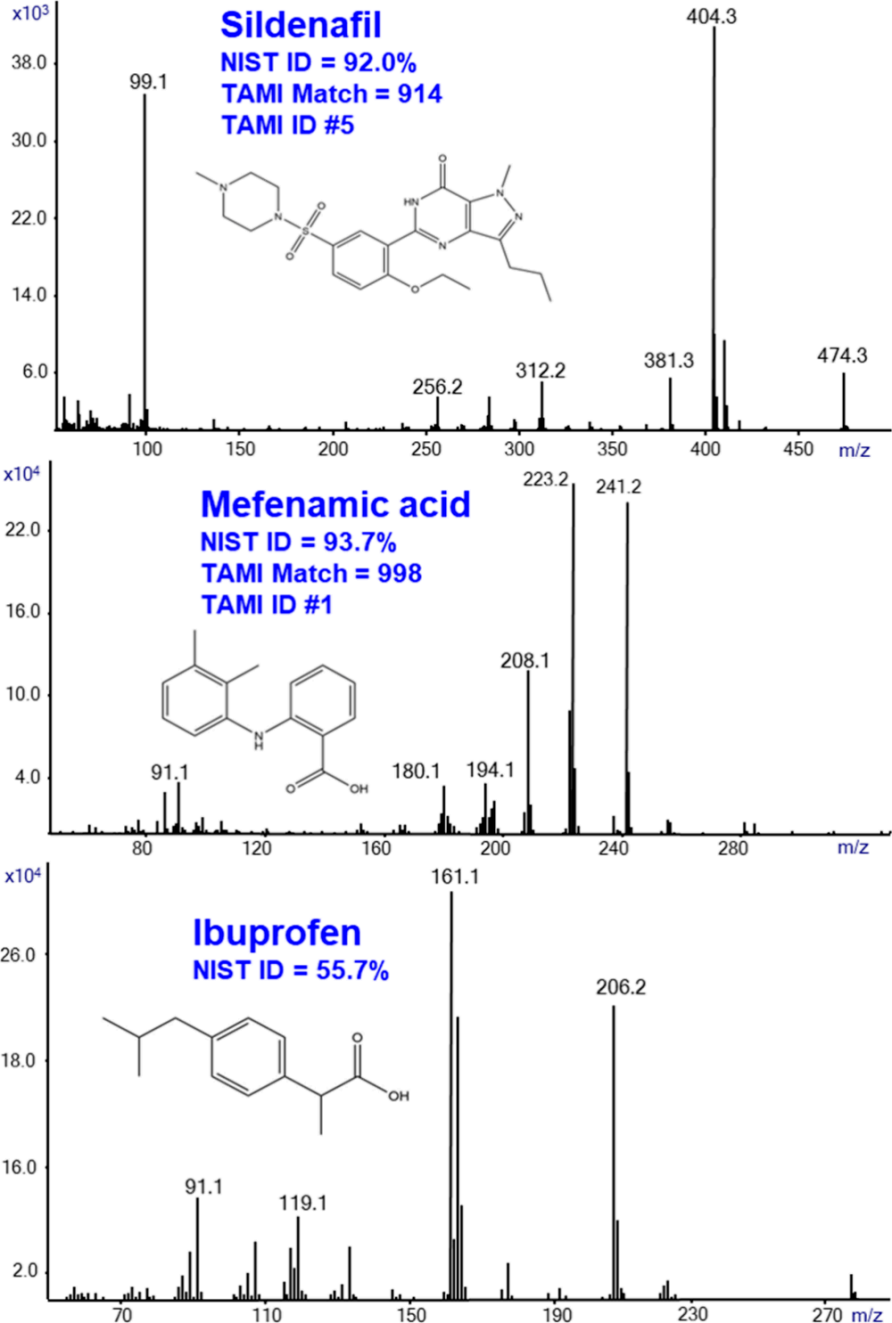
SIRTA Cold EI mass spectra of sildenafil, mefenamic acid, and ibuprofen. The active ingredients in these three drugs were obtained from medical pills that were dissolved in methanol. Each mass spectrum shows the NIST identification probability, and sildenafil and mefenamic acid also show the TAMI match and identification factors. The analysis was performed with inlet, GC oven, and transfer-line temperatures of 300 °C. The flow rate was 2.2 mL/min.

To explore the various analytical capabilities of SIRTA, we need to learn about its applicability to various types of compounds and applications. Figure 3 shows the application of SIRTA for drugs in medical pill formulations. It shows the generated SIRTA Cold EI mass spectra of sildenafil (upper mass spectrum), mefenamic acid (middle), and ibuprofen (bottom). The active ingredients were obtained from dissolved pill pieces in methanol. Sildenafil obtained from a Viagra pill exhibited a molecular ion in its Cold EI mass spectrum and a high NIST identification probability of 92%. The TAMI search confirmed the NIST identification and increased its probability to 99.8%, plus resulted in a TAMI match factor of 914. TAMI generated a list of elemental formulas that included sildenafil as #5, which is reasonably good. The Cold EI mass spectrum of mefenamic acid resulted in a high NIST identification probability of 93.7%, TAMI match of 998, and TAMI-rated elemental formula as #1 thereby exhibiting fully assured identification. Ibuprofen that was taken from an Advil capsule provided NIST identification probability of 55.7%, in part due to some other impurities in its mass spectrum. TAMI identification could not be obtained, as its molecular ion of *m*/*z* = 206 is just one mass unit from the abundant *m*/*z* = 207 PDMS impurity background that distorts the experimental isotope abundance. In SIRTA, unlike in GC-MS, mass spectral background subtraction does not work to better resolve the analyte from the noise.

**Figure 4:**
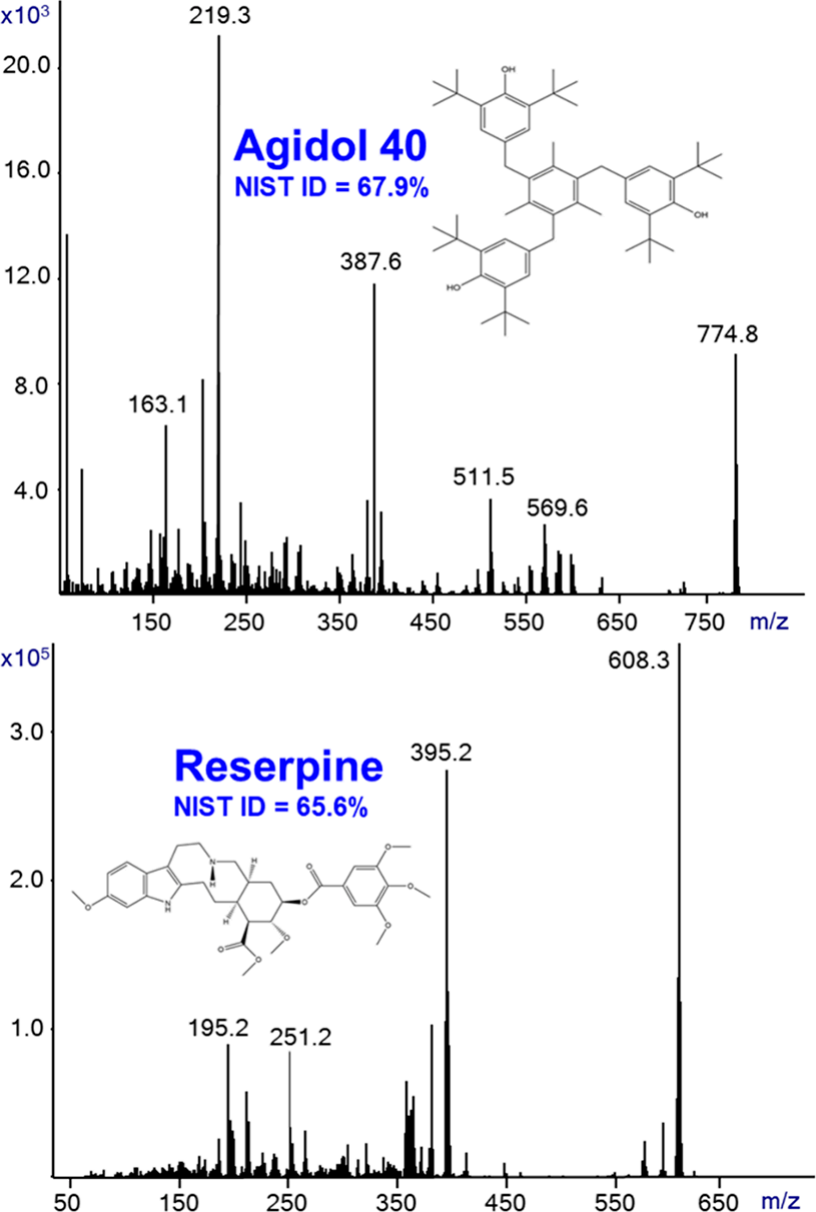
SIRTA Cold EI mass spectra of agidol 40 (C_54_H_78_O_3_) and reserpine (C_33_H_40_N_2_O_9_) as indicated by their names. NIST identification probabilities are also indicated. The analysis was performed with 280 and 320 °C inlet, GC oven, and transfer line temperatures for reserpine and agidol 40, respectively. The flow rate was 2.2 mL/min, and the mass spectral range was *m*/*z* = 50–800.

SIRTA serves as a better alternative to MS Probe, especially for the analysis of compounds that are incompatible using GC-MS with standard EI. Figure 4 shows the SIRTA Cold EI mass spectra of agidol 40 (C_54_H_78_O_3_) and reserpine (C_33_H_40_N_2_O_9_) in methanol. While reserpine serves as the LC-MS industry standard sensitivity specification compound, both reserpine and agidol 40 do not elute from a standard 30 m GC column or they decompose in the injector and/or column. The SIRTA of agidol 40 required a slightly higher injector, GC oven, and transfer line temperature of 320 °C for optimal analysis while reserpine required 280 °C due to partial decomposition at 300 °C. As shown in Figure 4, SIRTA provided highly informative Cold EI mass spectra that were readily identified using the NIST library (identification probabilities are indicated). Thus, SIRTA can serve as an alternative to MS Probe in the analysis of low volatility and thermally labile compounds yet with the important benefit of more informative Cold EI mass spectra with enhanced molecular ions.

**Figure 5:**
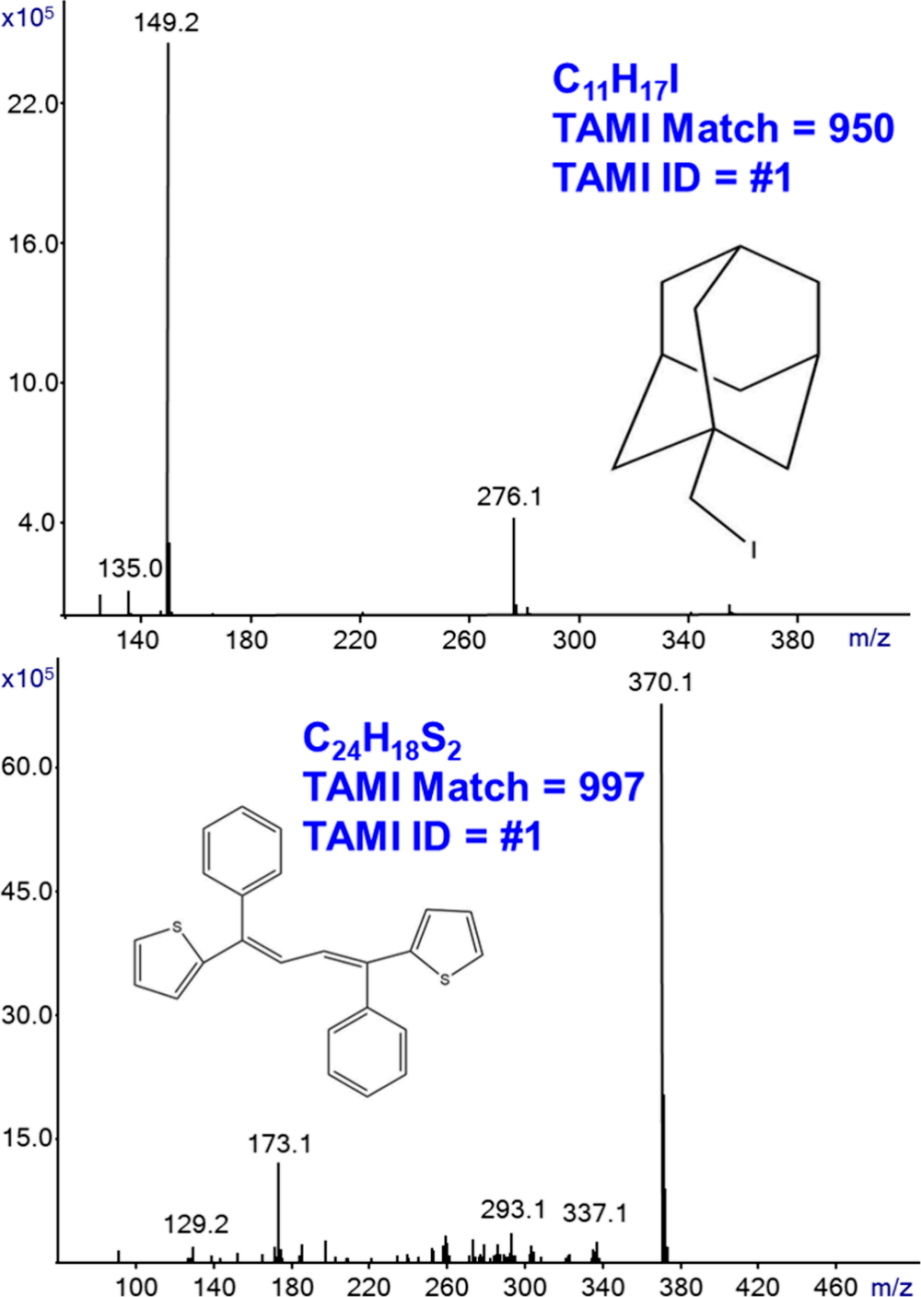
SIRTA Cold EI mass spectra of the indicated two synthesized organic compounds and their TAMI matches and numbers in the generated identification lists of elemental formulas. The analysis was performed with 160 and 240 °C inlet, GC oven, and transfer line temperatures. The flow rate was 2.2 mL/min, and the mass spectral range was *m*/*z* 50–500.

Another typical application of MS probe and/or the various ADI methods is the analysis of newly synthesized organic compounds that are usually too thermally labile for GC-MS with standard EI analysis but are properly analyzed by GC-MS with Cold EI.^28^ Accordingly, Figure 5 shows the generated SIRTA Cold EI mass spectra of two synthesized organic compounds, their TAMI matches, and their numbers in the TAMI-generated list of elemental formulas. Since these are newly synthesized compounds, they cannot be identified by the NIST or another library and thus the TAMI-based provision of elemental formula is essential. The C_11_H_17_I compound was analyzed from its NMR solution in CDCl_3_ just after its NMR analysis, the SIRTA Cold EI mass spectrum provided complete confirmation to the NMR data, and the TAMI software provided its elemental formula as commonly required for publication. The analysis was initially performed with 240 °C inlet, GC oven, and transfer line temperatures, but we suspected partial decomposition. Thus, Figure 5 data were obtained at 160 °C. The second compound of C_24_H_18_S_2_ was analyzed from its solid powder via touching the powder with a thin glass rod and dipping it in a vial with 0.7 mL of methanol that served for the SIRTA Cold EI analysis. Not unusual in Cold EI, C_24_H_18_S_2_ exhibited a dominant molecular ion and informative fragment ions. TAMI gave the correct elemental formula as #1 in the generated list. The TAMI software also provided the information that this compound must include two sulfur atoms.

**Figure 6:**
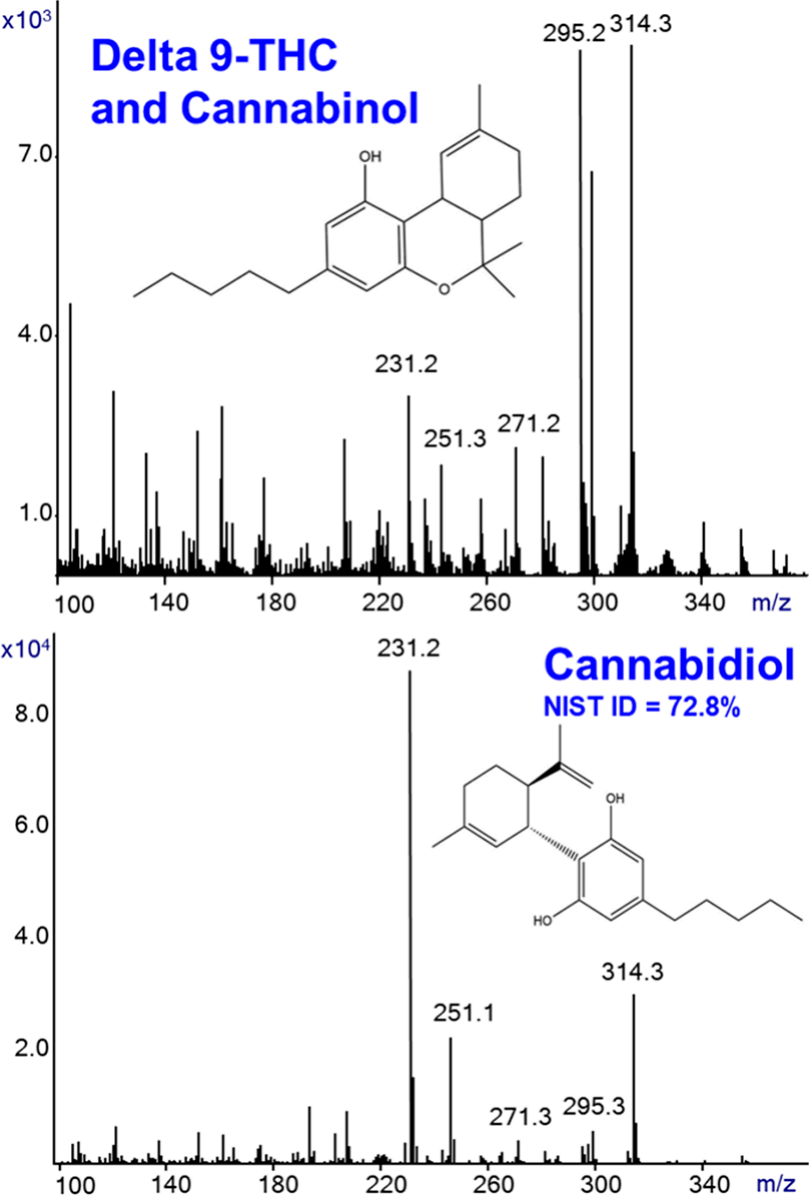
SIRTA Cold EI mass spectra of cannabis samples, including cannabis flower (upper) and EP-1 cannabis-based drug (bottom). The upper mass spectrum of the cannabis flower contained mainly delta 9-THC and cannabinol (CBN). The bottom mass spectrum of the cannabis-based drug contained mainly cannabidiol (CBD), and its NIST identification probability was 72.8%. The analysis was performed with 300 °C inlet, GC oven, and transfer line temperatures. The flow rate was 2.2 mL/min, and the mass spectral range was *m*/*z* = 50–500.

Another aspect of SIRTA that should be explored is its analysis of mixtures. Figure 6 shows the generated SIRTA Cold EI mass spectra of cannabis samples, including cannabis flower (upper) and cannabis-based drug (bottom). The cannabis dried flower was touched by a thin glass rod that was later dipped in a vial with methanol that served for its SIRTA with Cold EI. The cannabis-based drug was EP-1 for children with autism spectrum disorder, and the thin glass rod touched its liquid and was later dipped into a vial with methanol for its SIRTA with Cold EI. The upper mass spectrum in Figure 6 is of the cannabis flower, and it contains mainly delta 9-THC and cannabinol (CBN). The bottom mass spectrum of the EP-1 cannabis-based drug contains mainly cannabidiol (CBD) (plus 5% delta 9-THC). The NIST identification probability of CBD was 72.8%. Although the EP-1 drug is a mixture, its CBD content was dominant; thus, it could be identified by the NIST library.

**Figure 7:**
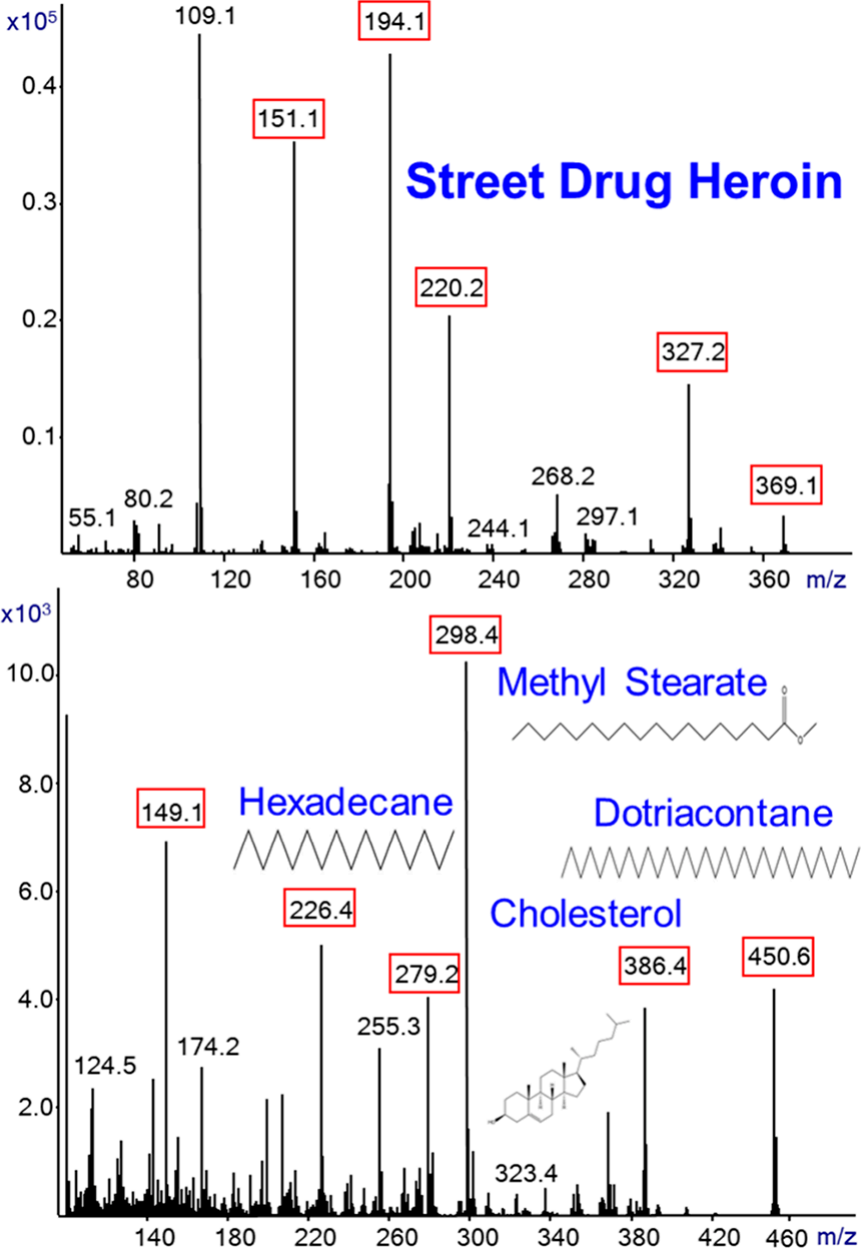
SIRTA Cold EI mass spectra of two mixtures. The upper mass spectrum is of a street drug heroin powder that was dissolved in methanol, which contains heroin, acetaminophen, caffeine, 6-monoacetylmorphine, and noscapine. The bottom mass spectrum is of a test mixture which contains 10 μg/mL each hexadecane, methyl stearate, cholesterol, dotriacontane, and some dioctyl phthalate in hexane. All molecular and other typical ions are marked with red boxes. The analyses were performed with 300 °C inlet, GC oven, and transfer line temperatures. The flow rate was 2.2 mL/min, and the mass spectral range was *m*/*z* = 50–500 and 90–500.

Figure 7 shows the generated SIRTA Cold EI mass spectra of two additional mixtures. The upper mass spectrum is of a street drug heroin powder that was dissolved in methanol, which contains mainly heroin, acetaminophen, caffeine, 6-monoacetylmorphine, and noscapine as described in their fast GC-MS with Cold EI analysis.^29^ Their molecular and other main ions are marked with red boxes. From the relatively low abundance of the *m*/*z* = 369.1 ions, we conclude that it is an old heroin mixture in which the heroin had partially degraded. The bottom mass spectrum is of a test mixture that serves to test the performance of Cold EI which contains 10 μg/mL each hexadecane, methyl stearate, cholesterol, dotriacontane, and a few μg/mL of dioctyl phthalate in hexane. Their molecular and other typical ions are marked with red boxes. As demonstrated, SIRTA with Cold EI can serve for the analysis of as low as 10 μg/mL of a few compounds and the use of hexane as solvent required the mass spectral initial ion to be *m*/*z* = 90 (the molecular ion of hexane is *m*/*z* = 86).

**Figure 8:**
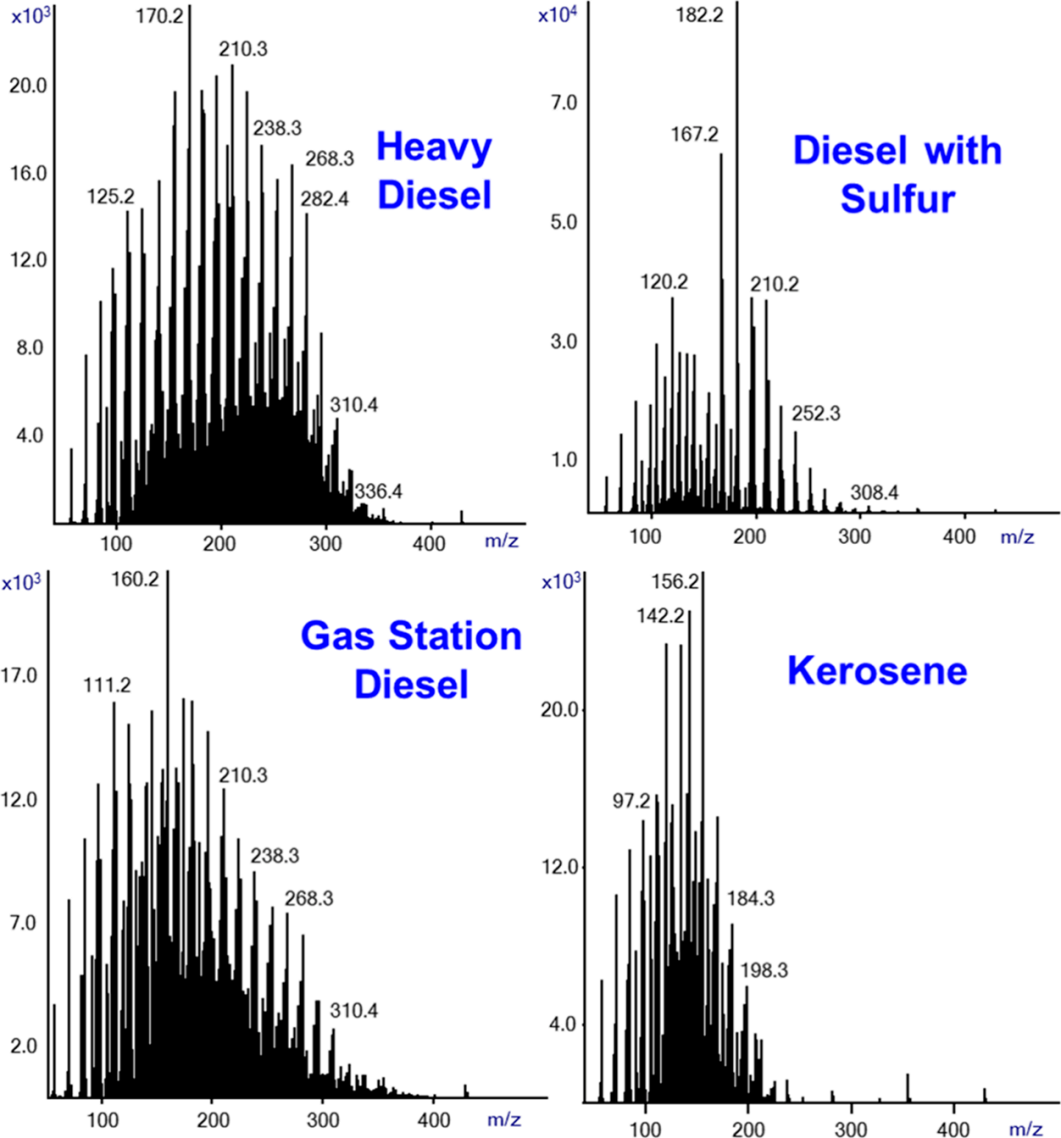
SIRTA Cold EI mass spectra of four types of fuels including heavy diesel and gas station diesel fuel from the local “PAZ” company, kerosene, and a diesel fuel with sulfur (sulfur standard). Each sample contained 0.2% fuel in methanol with 1 μL splitless injections. The analysis was performed at 300 °C inlet, GC oven, and transfer line temperatures. The flow rate was 2.2 mL/min, and the mass range was *m*/*z* = 50–500. The ion source was set to 13 eV electron energy.

We also explored the capability of SIRTA Cold EI analysis to provide valuable sample information in highly complex mixtures such as fuels via their Cold EI mass spectral fingerprints. Figure 8 shows the generated SIRTA Cold EI mass spectra of four types of fuels: (a) heavy diesel from a local PAZ company (upper left); (b) diesel fuel from a local PAZ gas station (bottom left); (c) kerosene from PAZ (bottom right); (d) diesel fuel with sulfur (sulfur standard) that was purchased from AccuStandard (upper right). The Cold EI ion source electron energy was reduced to 13 eV to achieve soft Cold EI mass spectra with mainly molecular ions for the various hydrocarbons. As shown in Figure 8, the complex mixture produces an overlay of many low eV EI mass spectra which complicates the identification of each component and obviously the library searching potential for identification is diminished. However, and as demonstrated, we obtained four different complex mass spectra that properly characterized the fuels via their unique mass spectral fingerprint. This is due to the unique capability of low electron energy Cold EI to provide mostly or nearly exclusively molecular ions for most hydrocarbons.

## 4 Conclusions

Real-time analysis with <1 min full analysis cycle time is highly beneficial. Flow injection analysis is available in LC-MS systems, simply by removing the LC column and replacing it with a low dead volume LC union. Flow injection provides an accessible and low-cost alternative to other forms of real-time analysis methods. However, while flow injection is available in MS of LC-MS systems it is not provided in MS of GC-MS systems, as the standard EI ion sources do not work well during solvent elution times in view of having high intraion-source space charge and possible damage to the filament. Furthermore, standard EI provides limited range of compounds that are amenable for analysis plus limited mass spectral information in view of the weakness or absence of molecular ions for a large portion of sample compounds. Thus, GC-MS with standard EI systems are in “solvent delay” mode during solvents elution times with the filament being off and no solvent injection for real-time analysis being used.

Standard EI ion sources were used during solvent elution such as in electron ionization LC-MS systems (EI-LC-MS) by various researchers, including the group led by Cappiello, as evidenced by their publications.^30,31^ EI provides a few unique benefits for LC-MS in comparison with ESI and APCI such as library-based identification including at the isomer level, ionization of nonpolar compounds, and the elimination of ion suppression effects. Notably, they have also implemented flow-injection analysis directly into the standard EI ion source without LC separation,^32−35^ and ref (35) shares similarities with this paper. However, in contrast to SIRTA with Cold EI, flow injection in EI-LC-MS systems required added hardware. Furthermore, flow injection with standard EI typically uses a low, sub-μL/min solvent flow rate (0.4 μL/min^33,34^) and thus can experience relatively long void time. We developed and employed electron ionization LC-MS with Cold EI^36^ either with separation or in its flow-injection analysis mode.^37^ It can serve for having better sensitivity than SIRTA due to its use of much higher solvent flow rate but it involves the addition of hardware and thus cost and complexity.

There are a few RTA methods and instruments such as MS probe, ChromatoProbe, and Open Probe Fast GC-MS that are effective in the provision of RTA with MS of GC-MS with in-vacuum ion sources. However, unlike flow injection they require the addition of hardware and cost. Thus, this paper is focused on the development of sample injection for real-time analysis (SIRTA) that does not add hardware and cost while being used in GC-MS with Cold EI and thus provides the simplest-to-use RTA method.

In this paper, we described how SIRTA is operated with Cold EI and showed its generated typical 1 min mass chromatograms via the injection of autosampler-based samples in vials while using 1 m of a 0.1 mm I.D. fused silica capillary to connect the injector and Cold EI MS transfer line with <0.3 s void time. We demonstrated the SIRTA operation with a range of drugs including low volatility such as reserpine and simple mixtures such as street drug heroin and cannabis up to and including complex mixtures such as diesel fuels. We also demonstrated the usefulness of Cold EI mass spectra that are characterized by exhibiting enhanced molecular ions and isomer-related fragment ions.

Accordingly, SIRTA combines flow injection MS of LC-MS features in the low-cost single quadrupole MS of GC-MS, avoiding hardware modifications and enabling the installation of a parallel GC column from a second injector for GC-MS analysis with separation alongside SIRTA. Thus, SIRTA adds another unique benefit to GC-MS with Cold EI of having MS Probe and flow injection capabilities with negligible added cost. Therefore, GC-MS with Cold EI can cost less than GC-MS with standard EI and MS Probe while providing much better sample information.

As a final conclusion, GC-MS with Cold EI is by far the best GC-MS technology with a broad range of benefits compared to standard EI,^18−20^ and SIRTA is another unique Cold EI benefit that improves its cost effectiveness.
